# Ultrasound-guided quadratus lumborum block versus caudal block for post-operative analgesia in childrens

**DOI:** 10.6026/973206300223663

**Published:** 2026-06-30

**Authors:** Monika Gandhi, Nidhi Sharma, Divya Rana, Shalini Jain

**Affiliations:** 1 Department of Anaesthesiology, Mahatma Gandhi Memorial Medical College, Indore, Madhya Pradesh, India

**Keywords:** Quadratus lumborum block (QLB), caudal block (CB), Post-operative analgesia, child, lower abdominal surgery, ultrasonography

## Abstract

Post-operative pain control in children undergoing lower abdominal surgery remains a concern because single-shot caudal analgesia has limited duration. Therefore, it is of interest to compare ultrasound-guided quadratus lumborum block and caudal block in 70 children, with 35 patients each in Group-QL and Group-C. Baseline age and gender distribution were comparable between the groups, with p = 0.580 and p = 0.550 respectively, while time to first rescue analgesia was significantly longer in Group-QL than Group-C (9.60 ± 0.59 h versus 3.37 ± 0.49 h, p = 0.001). Thus, ultrasound-guided quadratus lumborum block provided longer overall Post-operative analgesia, with significant difference in FLACC score at selected Post-operative time points.

## Background:

Effective Post-operative pain control in children remains a core perioperative goal because adequate analgesia is needed for comfort, physiological stability and smoother recovery after surgery [1]. Current pediatric pain guidance supports a multimodal perioperative approach and regional anesthesia is considered an important part of this pathway in children [2].Pediatric regional anesthesia has expanded beyond neuraxial methods toward ultrasound-guided peripheral and fascial plane techniques that may reduce opioid exposure and improve perioperative analgesia [3]. Caudal epidural block still remains one of the most commonly used regional techniques for infra-umbilical surgery in children because it is simple, safe and reliable in routine practice [4]. Single-shot caudal analgesia has limited duration, so alternative blocks and adjuvants are being evaluated for longer Post-operative pain relief in pediatric lower abdominal surgery [5]. Quadratus lumborum block is an ultrasound-guided truncal block for abdominal surgery and may provide somatic abdominal analgesia with wider thoracolumbar spread in children [ 6]. Recent pediatric discussion on fascial plane blocks suggests relatively better support for the transmuscular quadratus lumborum approach, though the evidence is still evolving [7]. In a large retrospective pediatric colorectal cohort, quadratus lumborum block showed high success with low pain scores, modest opioid use and no documented block related adverse events [8]. A report of favorable Post-operative analgesic efficacy and acceptable safety for quadratus lumborum block in pediatric surgical patients [9]. Pediatric data from lower abdominal laparoscopic surgery have also shown more effective Post-operative analgesia with ultrasound-guided quadratus lumborum block [10]. In direct comparison with caudal block ultrasound-guided quadratus lumborum block has shown longer Post-operative analgesia and higher parent satisfaction in children undergoing lower abdominal surgery [11]. Comparison of quadratus lumborum and caudal block suggested analgesic advantage for quadratus lumborum block with heterogeneity and need for further pediatric trials [12]. Ultrasound-guided abdominal blocks also highlighted that pediatric block outcomes need procedure-specific and outcome-specific interpretation rather than broad generalization [13]. A pediatric trial further showed longer analgesia with quadratus lumborum block than transverse abdominis plane (TAP) and ilioinguinal-iliohypogastric block in open inguinal hernia repair [14]. Another study showed lower FLACC scores at 6, 12 and 24 hours with quadratus lumborum block, with lower oral ibuprofen use, 6.7% versus 23.3% [15]. Lower pain scores, less analgesic consumption and longer time to first rescue analgesia with quadratus lumborum block than ilioinguinal-iliohypogastric block [1]. Therefore, it is of interest to compare ultrasound-guided quadratus lumborum block and ultrasound-guided caudal block for Post-operative pain relief in children undergoing lower abdominal surgery.

## Materials and Methods:

This prospective randomized controlled trial was conducted in the Department of Anaesthesiology, MGM Medical College and M.Y. Hospital, Indore over 12 months from May 2024 to May 2025 after Institutional Ethics Committee approval (EC/MGM/MAY 23/123). Seventy children aged 1-6 years undergoing elective lower abdominal surgery under general anaesthesia were included. Sample size was calculated using G*Power 3.1.9.2 with effect size 0.8, alpha 5%, power 90% and 10% dropout. The calculated sample size was 68, which was rounded to 70. Written informed consent was obtained from the legally authorized representative. Children with ASA physical status I or II, age 1-6 years, weight less than 20 kg and planned elective lower abdominal surgery were included. Children were excluded in case of refusal of consent, allergy to study drugs, bleeding diathesis, spinal deformity and local infection at the block site or neurological disorder. Patients were randomized into two groups of 35 each by odd-even method. Group QL received bilateral ultrasound-guided quadratus lumborum block with 0.25% bupivacaine 0.5 mL/kg on each side. Group C received ultrasound-guided caudal block with 0.25% bupivacaine 1 mL/kg. All children underwent standard pre-anaesthetic assessment and fasting. In the operating room, baseline heart rate, mean arterial pressure and SpO_2_ were recorded. Intravenous fluid was started. Premedication was given with glycopyrrolate and midazolam. Anaesthesia was induced with ketamine and succinylcholine and maintained with oxygen, nitrous oxide, sevoflurane and atracurium. After induction and before incision, the assigned block was performed under ultrasound guidance in lateral position under aseptic precautions. Hemodynamic parameters were recorded intraoperatively till the end of surgery. The primary outcome measure was time to first rescue analgesia. Secondary outcome measures included Post-operative FLACC pain score at predefined intervals, intraoperative and Post-operative hemodynamic parameters and adverse effects.

## Results and Discussion:

A total of 70 pediatric patients were included in the study, with equal allocation into the two study groups, as shown in Table 1. Group-QL comprised 35 patients (50.0%) and Group-C also comprised 35 patients (50.0%). Table 2shows the age-wise distribution of patients in both groups. In Group-QL, 7 patients (20.0%) were aged 1-2 years, 6 (17.1%) were 2-3 years, 6 (17.1%) were 3-4 years, 8 (22.9%) were 4-5 years and 8 (22.9%) were 5-6 years. In Group-C, 13 patients (37.1%) were aged 1-2 years, 5 (14.3%) were 2-3 years, 6 (17.1%) were 3-4 years, 5 (14.3%) were 4-5 years and 6 (17.1%) were 5-6 years. The mean age was 4.11 ± 1.47 years in Group-QL and 3.49 ± 1.69 years in Group-C. The difference in age distribution between the groups was not statistically significant (^2^ = 2.869, p = 0.580). Table 3 shows the gender-wise distribution of patients. In Group-QL, 27 patients (77.1%) were male and 8 (22.9%) were female; whereas in Group-C, 29 patients (82.9%) were male and 6 (17.1%) were female. The difference in gender distribution was not statistically significant (^2^ = 0.357, p = 0.550). Table 4shows the ASA grade distribution of patients. All patients in both groups belonged to ASA Grade I, with 35 patients (100.0%) in each group and no patient in any other grade. Here both groups were comparable with respect to baseline characteristics. In the present study, Group-QL and Group-C were comparable at baseline with no significant difference in age or sex distribution and all children belonged to ASA Grade I. This allowed a balanced comparison of Post-operative analgesic outcomes. Table 5 shows the Post-operative analgesic outcomes in both groups. Time to first rescue analgesia was significantly longer in Group-QL than in Group-C (9.60 ± 0.59 h vs 3.37 ± 0.49 h, p = 0.001). FLACC pain scores were comparable at 1, 2, 4, 5, 6, 10 and 12 hours. However, significant intergroup difference was seen at 3 hours and 8 hours. At 3 hours, the FLACC score was lower in Group-QL than Group-C (1.03 ± 0.62 vs 4.17 ± 1.38, p = 0.001), while at 8 hours it was higher in Group-QL (4.40 ± 1.38 vs 2.17 ± 0.66, p = 0.001). Thus, pain scores were not uniformly lower at all Post-operative intervals, but the overall duration of Post-operative analgesia remained longer in the QL group. No Post-operative adverse effects were observed in either group. Taken together, these findings suggest that ultrasound-guided quadratus lumborum block provided superior Post-operative analgesia to caudal block, mainly by prolonging analgesic duration and reducing overall analgesic requirement. Yadav *et al.* reported longer analgesia with QLB, 544.83 ± 60.22 minutes versus 350.67 ± 67.97 minutes, with lower analgesic requirement in the QL group [17]. Abdelbaser *et al.* however reported similar rescue analgesia rates between transversalis fascia plane block and QLB, 20.5% and 17.6% respectively [18]. Ralte *et al.* reported that both QLB and erector spinae plane block provided adequate analgesia during the first 24 hours after open pyeloplasty in children [19]. Rathod *et al.* found lower cortisol, glucose and fentanyl use with QLB, while Wageh *et al.* showed QLB gave analgesia comparable to epidural [20, 21]. Hung *et al.* reported in a network meta-analysis that QLB and TAP block had the longest time to first rescue analgesic in pediatric inguinal surgeries, while caudal block remained useful in orchidopexy [22]. Hofmann *et al.* also showed that caudal block had better intraoperative and early Post-operative efficacy in unilateral orchidopexy [23]. Based on the available tables the safest interpretation is that both groups were comparable before outcome assessment. In the present study, the main advantage of QL block over caudal block was the significantly prolonged duration of analgesia. This is clinically important in pediatric infra-umbilical surgeries, where longer pain relief may reduce early Post-operative analgesic requirement and improve recovery. Meta-analyses of pediatric ultrasound -guided blocks judge effectiveness mainly by pain events, rescue analgesia and complication profile [24]. A recent study also reported that quadratus lumborum block was more effective than caudal block for duration of post-operative analgesia consumption in paediatric abdominal surgery [25]. This supports the present findings. A recent protocol also suggests that this comparison remains clinically relevant and needs further updated evidence [26]. A supporting report on lower pain scores at 2, 4, 6 and 12 hours, longer time to first analgesic request, lower paracetamol consumption and less hypotension with quadratus lumborum block [27]. The study was conducted in a single centre with a relatively small sample size, which may limit generalizability. Only short-term perioperative outcomes were assessed, so longer-term analgesic effect and late complications could not be evaluated. This study adds comparative pediatric data on ultrasound-guided quadratus lumborum block and caudal block for lower abdominal surgery. It helps clarify whether quadratus lumborum block can provide a useful alternative for Post-operative pain relief in children.

## Conclusion:

Thus, ultrasound-guided quadratus lumborum block provided longer post-operative analgesia than caudal block. FLACC pain score differed significantly at selected postoperative time points, though not at all intervals. Ultrasound-guided quadratus lumborum block can be considered a safe and effective alternative to caudal block for post-operative analgesia in children undergoing lower abdominal surgery.

## Figures and Tables

**Table 1:** Distribution of pediatric patients by study group

**Group**	**Frequency (n)**	**Percentage (%)**
Group-QL	35	50
Group-C	35	50
Total	70	100

**Table 2:** Age-wise distribution of pediatric patients in Group-QL and Group-C

**Age (years)**	**Group-QL (n, %)**	**Group-C (n, %)**	**Chi-square**	**P-value**
1-2	7 (20.0%)	13 (37.1%)	2.869	0.58
2-3	6 (17.1%)	5 (14.3%)		
3-4	6 (17.1%)	6 (17.1%)		
4-5	8 (22.9%)	5 (14.3%)		
5-6	8 (22.9%)	6 (17.1%)		
Total	35 (100.0%)	35 (100.0%)		
Mean age	4.11 ± 1.47 years	3.49 ± 1.69 years		

**Table 3:** Gender-wise distribution of pediatric patients in Group-QL and Group-C

**Gender**	**Group-QL (n, %)**	**Group-C (n, %)**	**Chi-square**	**P-value**
Female	8 (22.9%)	6 (17.1%)	0.357	0.55
Male	27 (77.1%)	29 (82.9%)		
Total	35 (100.0%)	35 (100.0%)		

**Table 4:** ASA grade distribution of pediatric patients in Group-QL and Group-C

**ASA grade**	**Group-QL**(n, %)	**Group-C**(n, %)
Grade I	35 (100.0%)	35 (100.0%)
Other grades	0 (0.0%)	0 (0.0%)
Total	35 (100.0%)	35 (100.0%)

**Table 5:** Post-operative analgesic outcomes in Group-QL and Group-C

**Variable**	**Group-QL**	**Group-C**	**Test / p value**
Time to first rescue analgesia (h)	9.60 ± 0.59	3.37 ± 0.49	p = 0.001
FLACC score at 0 h	0.00 ± 0.00	0.00 ± 0.00	-
FLACC score at 1 h	0.14 ± 0.43	0.00 ± 0.00	p = 0.053
FLACC score at 2 h	1.02 ± 0.65	1.17 ± 1.50	p = 0.590
FLACC score at 3 h	1.03 ± 0.62	4.17 ± 1.38	p = 0.001
FLACC score at 4 h	1.49 ± 0.98	1.89 ± 1.71	p = 0.234
FLACC score at 5 h	1.46 ± 0.92	1.89 ± 1.72	p = 0.198
FLACC score at 6 h	1.57 ± 0.81	1.49 ± 0.85	p = 0.669
FLACC score at 8 h	4.40 ± 1.38	2.17 ± 0.66	p = 0.001
FLACC score at 10 h	2.51 ± 1.48	3.09 ± 0.98	p = 0.060
FLACC score at 12 h	1.91 ± 1.63	1.69 ± 1.30	p = 0.519
